# Environmentally Benign pSOFC for Emissions-Free Energy: Assessment of Nickel Network Resistance in Anodic Ni/BCY15 Nanocatalyst

**DOI:** 10.3390/nano13111781

**Published:** 2023-05-31

**Authors:** Margarita Gabrovska, Dimitrinka Nikolova, Hristo Kolev, Daniela Karashanova, Peter Tzvetkov, Blagoy Burdin, Emiliya Mladenova, Daria Vladikova, Tatyana Tabakova

**Affiliations:** 1 Institute of Catalysis, Bulgarian Academy of Sciences, 1113 Sofia, Bulgaria; margo@ic.bas.bg (M.G.); hgkolev@ic.bas.bg (H.K.); 2 Institute of Optical Materials and Technologies, Bulgarian Academy of Sciences, 1113 Sofia, Bulgaria; dkarashanova@yahoo.com; 3 Institute of General and Inorganic Chemistry, Bulgarian Academy of Sciences, 1113 Sofia, Bulgaria; tzvetkov@svr.igic.bas.bg; 4 Academician Evgeni Budevski Institute of Electrochemistry and Energy Systems, Bulgarian Academy of Sciences, 1113 Sofia, Bulgaria; b.burdin@iees.bas.bg (B.B.); e_mladenova@iees.bas.bg (E.M.); d.vladikova@iees.bas.bg (D.V.)

**Keywords:** pSOFC, BCY, impedance spectroscopy, hydrazine synthesis, metallic Ni, DRS, XPS, HRTEM

## Abstract

Yttrium-doped barium cerate (BCY15) was used as ceramic matrix to obtain Ni/BCY15 anode cermet for application in proton-conducting solid oxide fuel cells (pSOFC). Ni/BCY15 cermets were prepared in two different types of medium, namely deionized water (W) and anhydrous ethylene glycol (EG) using wet chemical synthesis by hydrazine. An in-depth analysis of anodic nickel catalyst was made aiming to elucidate the effect of anode tablets’ preparation by high temperature treatment on the resistance of metallic Ni in Ni/BCY15-W and Ni/BCY15-EG anode catalysts. On purpose reoxidation upon high-temperature treatment (1100 °C for 1 h) in air ambience was accomplished. Detailed characterization of reoxidized Ni/BCY15-W-1100 and Ni/BCY15-EG-1100 anode catalysts by means of surface and bulk analysis was performed. XPS, HRTEM, TPR, and impedance spectroscopy measurements experimentally confirmed the presence of residual metallic Ni in the anode catalyst prepared in ethylene glycol medium. These findings were evidence of strong metal Ni network resistance to oxidation in anodic Ni/BCY15-EG. Enhanced resistance of the metal Ni phase contributed to a new microstructure of the Ni/BCY15-EG-1100 anode cermet getting more stable to changes that cause degradation during operation.

## 1. Introduction

Global energy demand is expected to rise in the near future as a result of increasing urbanization and overpopulation worldwide. This vital problem cannot be met by the present electricity generation system based on depleting fossil fuel sources that emit greenhouse gases as a by-product. Increasing environmental drawbacks have forced scientific society to look for an alternative clean source [1,2,3]. The goal of European Union is diminishing greenhouse gas emissions and turn into climate neutral by 2050 [4].

Nowadays, the creation of energy by clean, efficient, and environmentally friendly ways turns to be one of the major challenges for researchers. In this regard, fuel cells (FCs) are recognized as a key energy technology with minimal impact on environment. FC represents energy conversion device that converts the chemical energy of a fuel directly to electrical energy and heat, from the chemical reaction between fuel (H^+^) and oxidizing agent (O_2_), without combustion. Among the types of FCs, a special position is assigned to solid oxide fuel cells (SOFCs) as a reliable, clean, and long-term source of energy. Operating at high temperatures from 800 to 1000 °C, SOFCs convert the chemical energy of the fuel, such as hydrogen or hydrocarbons, directly into electricity with efficiencies up to 60% in comparison with other FC types [1,2,5,6]. SOFCs are all solid-state electrochemical cells, modular, scalable, silent, fuel-flexible, and commonly working at high temperatures promote the electrochemical reaction at the electrodes in the presence of non-precious metal catalyst with good electricity generation efficiency. These characteristics make SOFCs applicable to a wide range of energy needs, both for stationary and portable power supplies [7,8,9]. A conventional SOFC consists of porous electrodes (anode and cathode) and a dense oxygen-ion-conducting electrolyte (ceramic or solid oxide material) forming a single cell. Between all of them, the main part of the ceramic fuel cell is the electrolyte. High temperature can provoke degradation of cell materials, mechanical stress, sintering of electrode, and a long start-up and shut-off period, etc., [10]. Therefore, a very important topic on the researchers’ agenda is to reduce the operating temperature of SOFCs to increase stability, to reduce the price by using cheaper components, to reduce startup and shutdown durations and to improve cell’s tolerance to thermal cycling, thus providing a more cost-effective alternative to conventional high-temperature SOFC [11,12].

The intensive scientific and industrial development led to the discovery of proton-conducting solid oxide fuel cells (pSOFCs). The main features of pSOFCs are high proton conductivity, low activation energy, a wide variety of the sealing and interconnection materials, potential to work at intermediate temperatures (400–600 °C) for long operational life time, etc., [10,12,13]. Proton conductors are practicable electrolytes at intermediate temperatures because protons migrate more easily than oxygen ions at 400–600 °C thus relieving SOFCs technological problems [14].

Oxygen-deficient ceramic oxides, in particular with perovskite type structure as BaCeO_3_- and BaZrO_3_-based oxides are reported as most suitable solid electrolyte materials for intermediate temperature fuel cells due to higher proton conductivity and lower activation energy compared to a typical oxygen ion conducting electrolyte [5,13,15]. Since perovskite materials produce the highest proton mobility in oxides, they are still the most promising proton conducting electrolytes. Among them, barium cerate (BaCeO_3_) ceramics exhibit an exceptional proton conductivity [16]. Proton conductivity is affected by proton transport, chemical stability of the electrolyte, formation of defects into the perovskite structure, and their distribution in the crystalline lattice. Targeting to improve the main features of BaCeO_3_ led to the conclusion that dopants with low ionic charge (higher ionic size) can enhance proton conductivity. For example, doping with acceptor (Y^3+^) compared to the lower ionic size of Ce^4+^ ions lead to the creation of oxygen vacancies, which play an essential role in the formation of mobile protons [5,13].

Yttrium-doped barium cerate at Ce^4+^ sites (BaCe_1−x_Y_x_O_3−δ_), commonly noted BCY, is considered the main electrolyte for pSOFC at a lower temperature due to high proton conductivity, high electronic conductivity, and excellent chemical stability under reduced fuel cell environment over a wide temperature range [12,17]. BCY is a classical proton-conducting electrolyte, which may be applied in two different functional layers of pSOFC: as proton conducting electrolyte and metal-cermet anode.

Inspection of the literature suggests that nickel is the most widely applied metal for incorporation in the anode ceramic matrix to form cermet anodes. The choice of Ni is due to excellent catalytic activity for hydrogen oxidation in the intermediate temperature range, high electrical conductivity, thermal compatibility with other cell components, low cost as well as performance similar to that of the precious metal-based catalysts, thus offering significant cost savings for the production of inexpensive and environmentally friendly energy systems [18]. To ensure sufficient current collection, Ni content is usually over 35 vol. % to form a percolation path for electron transport [19]. Proton-conducting ceramic and Ni form an anode cermet composite material. The presence of proton conductor in the anode is of great importance because it inhibits high-temperature coarsening and grain growth of the metallic Ni particles as well as plays a crucial electrocatalytic role for creation of additional reaction sites where anode reaction occurs. The proton conductor ensures a conductivity network for H^+^ ions and extends the triple-phase boundary (TPB) length resulting in electrode performance improvement [18]. Accordingly, a suitable dispersion state of the Ni particles in the anode is obligatory.

Different aspects related to the Ni-based anode catalyst preparation techniques are referred to commercial mechanical mixing, combustion method, co-precipitation, spray pyrolysis, sol-gel, electroless deposition, pulsed laser irradiation-assisted synthesis, etc. The impact of these synthesis methods on cell performance, microstructure, morphology, Ni grain size and surface area, role, and effect of the proton conductor, etc., have been extensively examined and debated in many scientific reviews and numerous papers [2,3,9,20,21,22,23]. Only the solid-state reaction (mechanical mixture of oxide powders) and the combustion method have been investigated from the industrial point of view. However, the solid-state reaction is an expensive method involving extreme experimental conditions, which leads to the production of coarse and inhomogeneous materials, while metal ceramics with pure crystalline phases cannot be easily obtained by applying the combustion method [24].

Rational design of nanocatalysts for pSOFC is an attractive approach to providing clean air and green, climate-neutral energy in line with decarbonization strategies for achievement of environmental health and safety.

In this respect, the existence of well-dispersed nanosized metal Ni particles in the cermet will stimulate generation of numerous catalytic sites for hydrogen adsorption that is considered an effective solution to improve electrode reaction kinetics; enhancement of the anode performance, and improvement of redox cycling stability [11].

Wet chemical synthesis technique or liquid phase synthesis is well-known as a simple and economically profitable procedure for preparation of a wide range of nanomaterials offering a strict control on size, morphology, and structure as well as reproducibility of nanomaterial preparation. Being a promising alternative, this method can be useful for synthesizing almost all metals, metal chalcogenides, and metal oxides [25].

Wet reduction of Ni^2+^ ions with hydrazine (N_2_H_4_) in anhydrous environment is reported as a successful synthetic route for synthesis of nickel nanoparticles [26]. This method allows easily controllable morphology of the nickel powders, particle size, and agglomeration degree through the reaction parameters—solvent composition, nucleation agent, surfactant, etc., [26,27,28,29,30].

In our previous studies, we reported for the first time the hydrazine wet chemical reduction route in the pSOFC field as an approach for preparation of Ni/BCY15 anode catalyst [30,31]. In this connection, the following synthesis conditions were developed: order of solution addition, type of alkaline solution, mixture of NaOH and Na_2_CO_3_ concentrations, and pH value. Low temperature wet reduction of Ni^2+^ ions by hydrazine offers direct incorporation of metallic Ni into the anode ceramic matrix of yttrium-doped barium cerate, BaCe_0_._85_Y_0_._15_O_2_._925_ (BCY15). Based on this parallel evaluation of the applied deionized water (W) and anhydrous ethylene glycol (EG) media, it was established that both as-synthesized catalyst samples contain nanosized metallic Ni particles. It was found that Ni/BCY15-EG exhibits lower resistance values than Ni/BCY15-W thus demonstrating improved electrochemical performance in comparison with a cermet synthesized in water [30,31]. Applied accelerated stress test of Ni cermet synthesized in EG medium estimating degradation of the metallic Ni phase during 6 reduction/oxidation cycles indicated that the metallic Ni phase in Ni/BCY15-EG anode is more stable to reoxidation compared to a very fast degradation of commercial Ni/BCY anode prepared by mechanical mixing procedure [31]. It was stated that the application of the wet-chemical synthesis route in EG environment provides formation of new microstructure in the Ni/BCY15 pSOFC anode that is more tolerant to redox cycling [31]. However, there are no literature data about analysis of the reasons for stability of the metal nickel obtained by wet chemical reduction route applying combined reduction capability of hydrazine and ethylene glycol.

With the purpose to upgrade our previous research, pointed to insights on the strength of interface between hydrazine-originating metallic Ni and cerium from the BCY15 matrix, the target of the current work is to elucidate the effect of the stronger Ni^0^–Ce^3+^ bond formed during the wet chemical reduction synthesis in anhydrous ethylene glycol environment on the change in Ni/BCY15 catalyst microstructure during the process of anode tablet preparation by high temperature sintering, an obligatory stage in the technological cycle. On purpose reoxidation upon high-temperature treatment (1100 °C for 1 h) in air ambience of as-synthesized reduced Ni/BCY15-W and Ni/BCY15-EG anodic catalysts was accomplished aiming an in-depth analysis of the resistance of nano-scaled metallic Ni phase on transformation to NiO during cermet anode fabrication. Comparative examination was applied for detailed characterization of the reoxidized reduced catalysts by surface and bulk analysis with several physicochemical methods, such as PXRD, XRF, DRS, XPS, TEM, and TPR. Electrochemical analysis by impedance spectroscopy was also performed.

The novelty of this investigation was to find new evidence for stability of the hydrazine-originating metallic Ni as a beneficial factor for optimized degradation of Ni cermet for strong pSOFC longevity.

## 2. Materials and Methods

### 2.1. Materials

Yttrium-doped barium cerate powder BaCe_0_._85_Y_0_._15_O_2_._925_ (BCY15), supplied by Marion Technologies, was fabricated by auto-combustion method and applied as the anode ceramic matrix. Before synthesis, the BCY15 powder was thermally pretreated at 1100 °C for 2 h. Nickel chloride hexahydrate (NiCl_2_·6H_2_O), hydrazine monohydrate (99+% N_2_H_4_·H_2_O), sodium hydroxide (NaOH), and anhydrous sodium carbonate (Na_2_CO_3_) were procured by Alfa Aesar (Ward Hill, MA, USA). Anhydrous (99.8%) ethylene glycol (EG) was acquired by SIGMA-ALDRICH. All reagents were of “pro analyze” purity grade and were used as received without further purification.

### 2.2. Synthesis

Two Ni/BCY15 anode catalysts with the same composition of NiO/BCY15 = 44.4/55.6 (volume ratio) were synthesized by hydrazine low temperature wet chemical reduction of NiCl_2_ using two types of medium: aqueous—deionized water and anhydrous—ethylene glycol environment. The applied preparation procedure provided direct incorporation of metallic Ni into the anode ceramic matrix BCY15. EG use ensured an anhydrous environment to act as a further reducing agent. Thus, EG promoted a deeper nickel reduction and avoids partial decomposition of the BCY 15 ceramic matrix due to high affinity to water. Detailed description of the preparation procedures can be found in Ref. [30].

The catalyst samples, which were subjected to extreme reoxidation at 1100 °C, are denoted as Ni/BCY15-W-1100 and Ni/BCY15-EG-1100.

### 2.3. Sample Characterization Methods

#### 2.3.1. Powder X-ray Diffraction (PXRD)

Powder X-ray diffraction (PXRD) technique was performed to establish phase composition of the catalyst samples after extreme reoxidation. The patterns were recorded at room temperature in the 5–100° 2θ range on a Bruker D8 Advance diffractometer (Bruker-AXS, Karlsruhe, Germany) using CuKα radiation (λ = 0.15406 nm) and LynxEye detector (Bruker-AXS, Karlsruhe, Germany) operated at U = 40 kV and I = 40 mA. Crystalline phase identification was based on International Centre for Diffraction Data (ICDD) powder diffraction files. A semi-quantitative analysis (as wt.%) was carried out using Diffrac.Eva V4 program (Bruker-AXS, Karlsruhe, Germany).

#### 2.3.2. X-ray Fluorescence Spectrometry (XRF)

X-ray fluorescence spectrometry (XRF) was used for quantitative determination of material’s chemical composition. It was accomplished on a Fischerscope XDAL instrument, Software WinFTM BASIC including PDM (Sindelfingen, Germany). XRF analysis provided a fast qualitative and quantitative elemental analysis at concentration levels from sub parts-per-million (ppm) to 100%.

#### 2.3.3. UV-Vis Diffuse Reflectance Spectroscopy (DRS)

UV-Vis spectra were recorded on a Thermo Scientific Evolution 300 spectrophotometer (Thermo Electron Scientific Instruments LLC, Madison, WI, USA) equipped with a Praying Mantis Diffuse Reflectance Accessory. Registered diffuse-reflectance (DR) spectra were transformed into absorbance mode by applying Kubelka-Munk equation: F(R) = (1 − R)^2^/2R.

#### 2.3.4. X-ray Photoelectron Spectroscopy (XPS)

X-ray photoelectron measurements were carried out on an ESCALAB MkII (VG Scientific, now Thermo Fisher Scientific, Waltham, MA, USA) electron spectrometer, equipped with twin anode MgKα/AlKα non-monochromated X-ray source with excitation energies of 1253.6 and 1486.6 eV, respectively. Instrumental resolution was about 1 eV. Electrostatic sample charging was compensated through energy scale calibration by normalizing the C1s line of adventitious hydrocarbons to 285.0 eV. The measured spectra were processed with SpecsLab2 CasaXPS software version 2.3.25PR1.0 (Casa Software Ltd., Teignmouth, UK) including the subtraction of X-ray satellites and Shirley-type background [32]. The relative concentrations of the different chemical species were determined based on normalization of the peak areas to their photoionization cross-sections as calculated by Scofield [33].

#### 2.3.5. Transmission Electron Microscopy (TEM)

The morphology of the studied samples was revealed by Jeol JEM 2100 (JEOL, Tokyo, Japan) high-resolution transmission electron microscope at 200 kV accelerating voltage in conventional mode. Sample phase composition was registered by diffraction methods as selected area electron diffraction (SAED) and high-resolution transmission electron microscopy (HRTEM). Match software 3.13 Version (Crystal Impact, Bonn, Germany) and Crystallographic Open Database (COD) was used for phase identification.

#### 2.3.6. Temperature-Programmed Reduction (TPR)

Hydrogen temperature-programmed reduction (TPR) of the extreme reoxidized catalysts was carried out in the measurement cell of a SETARAM model DSC-111 differential scanning calorimeter (SETARAM, Caluire, France). The temperature was linearly increased from 25 to 700 °C at a heating rate of 10 °C min^−1^. The TPR experiments were accomplished by a gas mixture of 10% H_2_ in Ar at a flow rate of 20 cm^3^ min^−1^. To avoid mass transfer and temperature control limitations, the experimental conditions were chosen to agree with the criteria recommended by Monti and Baker [34]. Deconvolution of the original TPR profiles using the Magic Plot Pro Ver 2.5.1. (Magicplot Systems, Saint Petersburg, Russia) software package by combining only Gaussian functions was done.

### 2.4. Electrochemical Measurements

Electrochemical impedance spectroscopy was carried out on IVIUM—CompactStat e10030 (Alvatek Ltd, Tetbury, England) in the frequency range 1–0.1 Hz with density 5 points/decade at room temperature. To take into account the geometry of measured samples and to compare the two materials, the resistivity was calculated by the equation: r = R.A/l, where R is the measured resistance, A is the cross-sectional area of the measured samples, and l is their thickness.

## 3. Results and Discussions

### 3.1. Coloration of the Forced Reoxidized Catalysts

**Figure 1:**
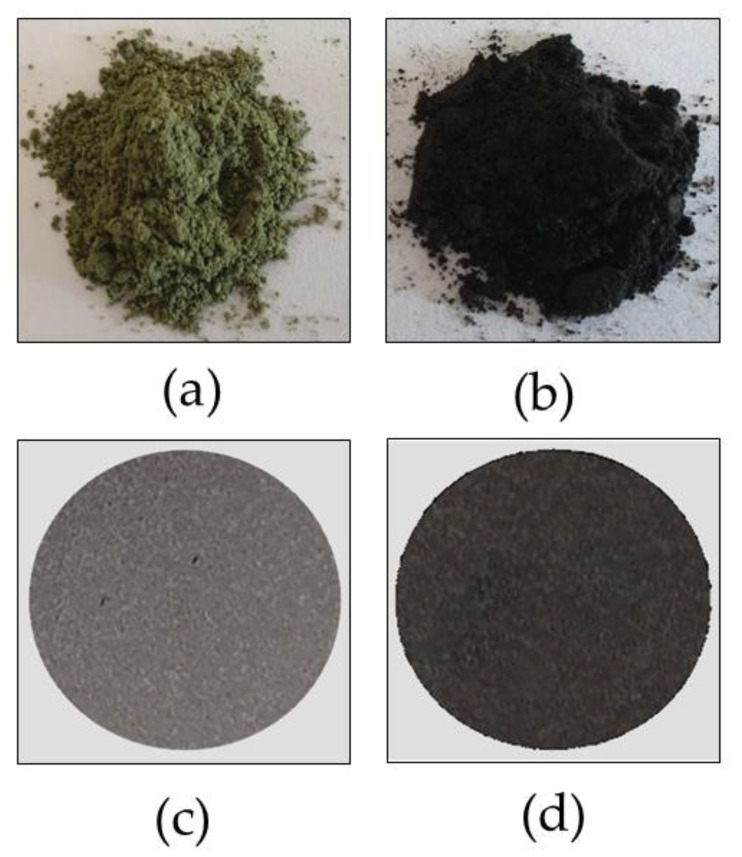
Photographs of anode catalysts: (**a**) Ni/BCY15-W-1100 and (**b**) Ni/BCY15-EG-1100, and anode pressed tablets: (**c**) Ni/BCY15-W-1100 and (**d**) Ni/BCY15-EG-1100.

Forced reoxidation at 1100 °C resulted in color differences of the samples, namely green for Ni/BCY15-W-1100 (Figure 1a) and black for Ni/BCY15-EG-1100 (Figure 1b). Color variations were preserved after making the pressed tablets (Figure 1c,d).

It is important to note that the as-synthesized reduced Ni/BCY15-W and Ni/BCY15-EG catalysts were black colored due to metallic Ni deposition over the BCY15 matrix. Sample sintering at 1100 °C in an oxidizing environment (air) should indicate a progressive oxidation of the metallic Ni and finally obtain pale green NiO samples of large particle size. Obviously, the black color of the as-synthesized reduced Ni/BCY15-EG sample did not change during forced reoxidation at a high temperature.

Upon studying NiO as a photocathode in p-type dye-sensitized solar cells, Renaud et al. [35] also obtained black colored NiO. They attributed this tint to the existence of a small amount of metallic Ni within the NiO and not to the presence of mixed valence Ni^2+^/Ni^3+^ species as commonly suggested in the literature.

As aforementioned our efforts were focused on a search for metallic Ni by studying the structure, surface electronic state, microstructure, and reducibility of the differently colored reoxidized Ni/BCY15 anode catalysts. Next, we attempted to recognize the contribution of Ni metal presence in NiO environment to the electrochemical behavior of the Ni/BCY15-W-1100 and Ni/BCY15-EG-1100 anodes.

### 3.2. Powder X-ray Diffraction (PXRD)

**Figure 2:**
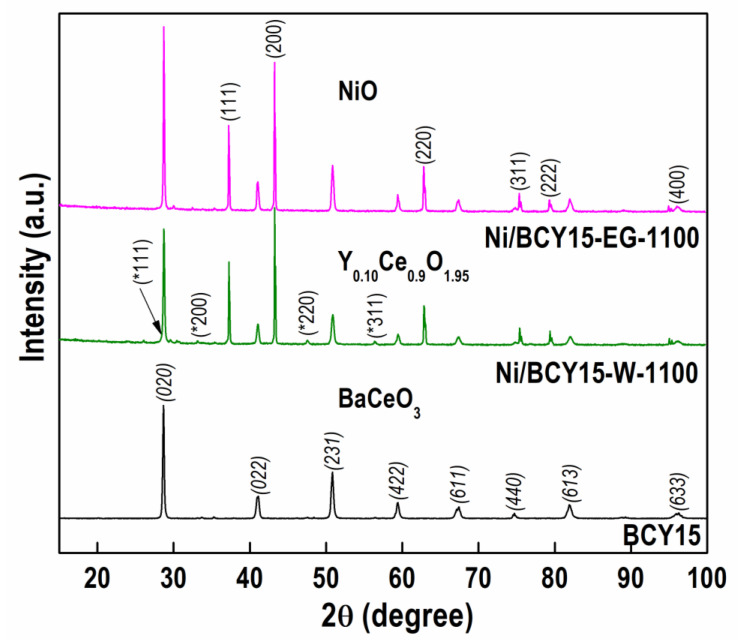
PXRD patterns recorded for BCY15 and reoxidized Ni/BCY15-W-1100 and Ni/BCY15-EG-1100 samples. The main diffraction peaks of BCY15 are marked in italics, those of NiO are regularly denoted, while additional asterisk points out the main diffraction peaks of the Y_0_._1_Ce_0_._9_O_1_._95_ phase.

The structural features of reoxidized Ni/BCY15 catalysts were evaluated by means of PXRD analysis (Figure 2). For comparison, a pattern of BCY15 powder is also shown.

The diffraction patterns of BCY15 consist of reflections located at the angles typical of orthorhombic perovskite isostructural with BaCeO_3_ (ICDD-PDF file 00-022-0074) and yttrium-doped analogue BaCe_0_._9_Y_0_._1_O_2_._95_ (ICDD-PDF file 01-081-1386). The presence of single perovskite phase in BCY15 was confirmed by comparison of the BCY15 crystal lattice parameter data with those of standard BaCeO_3_ and BaCe_0_._9_Y_0_._1_O_2_._95_ (Table 1).

In addition, PXRD patterns that display reflections indexed as (111), (200), (220), (311), and (222) of cubic NiO phase (ICDD-PDF file 00-047-1049) were also registered with Ni/BCY15-W-1100 and Ni/BCY15-EG-1100. A pure nickel oxide phase with very sharp peaks typical of large grains and a high crystallinity degree was formed.

Comparison indicates that reoxidized samples display diffraction lines of BCY15 and NiO phases with different crystallinity. PXRD analysis (Figure 2) revealed better-formed reflections of BCY15 perovskite structure in Ni/BCY15-EG-1100 that is in accordance with a larger BCY15 crystallite size of 509 nm vs. 389 nm in Ni/BCY15-W-1100 (Table 1). It is clearly seen that NiO reflections are better organized in the Ni/BCY15-EG-1100 catalyst than those with Ni/BCY15-W-1100. Different sample crystallinity is also evidenced by the estimated mean crystallite size of the NiO phase, being 300 and 350 nm with Ni/BCY15-W-1100 and Ni/BCY15-EG-1100, respectively (Table 1). The data indicate a higher crystallinity of the sample prepared in ethylene glycol medium.

Besides the diffraction peaks of NiO and BCY15 as prevailing phases, additionally a few reflections of lower intensity of a cubic Y_0_._10_Ce_0_._90_O_1_._95_ phase (ICDD-PDF file 01-075-0174) were disclosed in the PXRD patterns of Ni/BCY15-W-1100.

No peaks indexed to metallic Ni were detected in both samples probably due to quite low amount.

Based on PXRD examination, a semi-quantitative analysis was performed aiming to estimate the amount of registered phases in the extreme reoxidized catalysts (Table 2). The results show equal concentrations of NiO and similar values for BCY15. A relatively lower concentration of BCY15 in Ni/BCY-W-1100 is attributed to the presence of Y_0_._10_Ce_0_._90_O_1_._95_ phase.

The absence of yttrium-cerium oxide phase in Ni/BCY15-EG-1100 catalyst is evidence for the preparation of pure anode cermet.

The PXRD analysis demonstrates that the presence of metallic Ni in the Ni/BCY15-EG-1100 catalyst could not be identified.

### 3.3. X-ray Fluorescence (XRF) Spectrometry

Elemental bulk analysis was also performed by XRF. The composition was determined by measurements into three spots in each of the reoxidized catalyst, displayed in Table 3.

After applied reoxidation, the first observation is consistent with a Ni/BCY15-EG-1100 highly homogenous structure of the reoxidized catalysts in accordance with data from semi-quantitative PXRD calculation data.

### 3.4. Surface Electronic State Analysis

Surface electronic state was estimated by UV-vis diffuse reflectance spectroscopy and X-ray photoelectron spectroscopy measurements.

#### 3.4.1. Diffuse Reflectance Spectroscopy (DRS)

Diffuse reflectance spectroscopy was used to analyze surface coordination and electronic state of the components in BCY15 and Ni-cermets in the UV (200–400 nm) and Vis (400–800 nm) regions.

**Figure 3:**
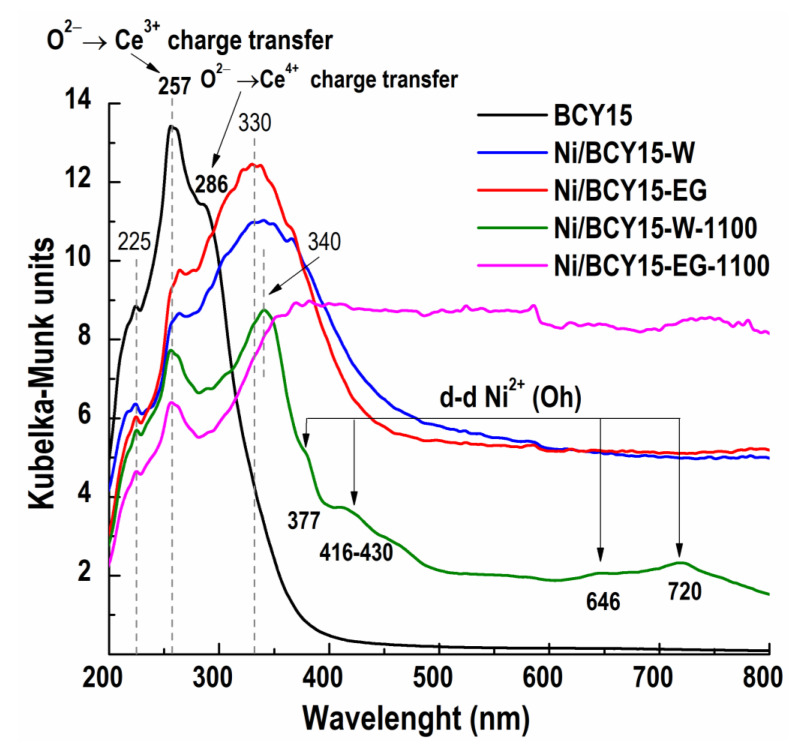
UV-Vis spectra of BCY15, as-synthesized reduced Ni/BCY15-W and Ni/BCY15-EG and reoxidized Ni/BCY15-W-1100 and Ni/BCY15-EG-1100 catalysts.

Based on the literature data [36,37,38,39,40,41], the results of diffuse reflectance electronic structure measurements of the bare BCY15 presented in Figure 3 are classified as absorption bands of ligand-to-metal charge transfer emissions of yttrium and cerium appearing in the UV region:

- O^2−^ → Y^3+^, a charge transfer at 225 nm;
- O^2−^ → Ce^3+^, a charge transfer at 257 nm;
- O^2−^ → Ce^4+^, a charge transfer at 286 nm.

Identification of Ce^3+^ defects formed at partially replaced Ce^4+^ host cations by incorporated Y^3+^ ions into the BCY15 structure has been reported by the analysis of the bulk BCY15 matrix by electron paramagnetic resonance (EPR) spectroscopy in a previous paper [31]. Ce3d level deconvolution confirmed the coexistence of Ce^4+^ and Ce^3+^ oxidation states on the BCY15 surface.

In spite of the black color, as-synthesized reduced Ni/BCY-W and Ni-BCY-EG samples were also investigated to gain reference information. Deposition of metallic Ni provoked decreased light absorbance and a slight red shift of O → Ce^3+^ charge transfer band with both catalysts related to the BCY15 spectra, which is indication for interaction between Ni^0^ and Ce^3+^ sites as established by XPS and EPR [31]. Bands in the range 290–370 nm are ascribed to ligand-to-metal charge transfer of O^2−^ to Ni^2+^ in Ni(OH)_2_ [42,43]. The presence of Ni^2+^ ions on the surface was also registered by XPS analysis of as-synthesized reduced Ni/BCY-W and Ni-BCY-EG cermets. This is attributed to predicted formation of surface Ni(OH)_2_ owing to surface passivation of the formed metallic Ni because of washing by water and air drying, being steps in hydrazine reduction procedure. No absorption bands due to d-d transitions of Ni^2+^ ions were detected in the Vis DR spectra due to the black color of both as-synthesized reduced catalysts.

Upon initial inspection of Figure 3, the light absorbance in the visible region conforms to the black color of Ni/BCY-EG-1100 cermet after reoxidation treatment at extreme conditions of high temperature and air atmosphere (Figure 1b). A red shift of the DR spectra was also registered. Both observations testify the presence of residual metallic Ni being undetectable by PXRD. Identified extra absorption at 380 nm results from changes and surface defects originated in the structure of thermally treated Ni/BCY-EG-1100 cermet as compared to as-synthesized reduced state.

Concerning Ni/BCY-W-1100 surface electronic state of dark green colored sample after reoxidation of the metallic Ni (Figure 1a), the DR spectra contained absorption bands above 300 nm proving a Ni^2+^ coordination state:

- O^2−^ → Ni^2+^, a charge transfer at 340 nm;
- d → d transitions of Ni^2+^ (d8) ions in octahedral (Oh) coordination in NiO lattice: spin-allowed transitions at 377 and 720 nm (3A_2g_(F) → 3T_1g_(F) and 3A_2g_(F) → 3T_1g_(P)) and spin forbidden transitions at 413 and 646 nm (3A_2g_(F) → 1E_g_(D) and 3A_2g_(F) → 1T_2g_(D)) [44,45,46].

A band characteristic of O^2−^ → Ce^3+^ charge transfer at 257 nm and a very low intense O^2−^ → Ce^4+^ charge transfer transition band at 290 nm were identified in the spectra of both treated cermets having lower absorption that can be explained by changed electron environments after formation of NiO.

The surface electronic structure of Ni/BCY15-W-1000 and Ni/BCY15-EG-1000 cermets was also evaluated by means of X-ray photoelectron spectroscopy.

#### 3.4.2. X-ray Photoelectron Spectroscopy (XPS)

**Figure 4:**
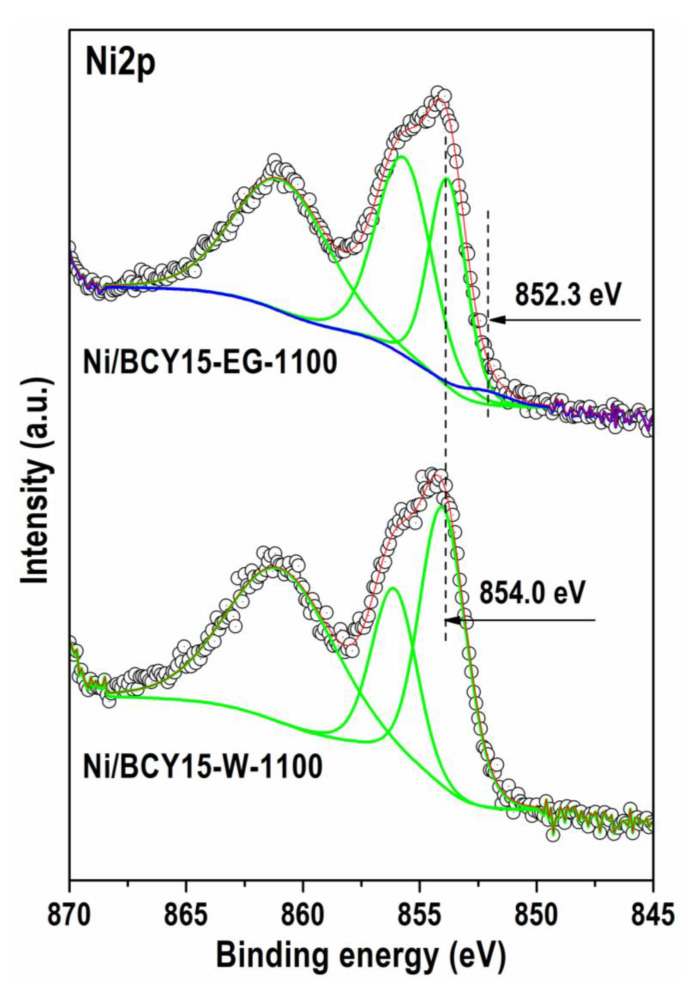
Ni2p photoelectron region of the reoxidized Ni/BCY-W-1100 and Ni/BCY-EG-1100 catalysts.

The Ni2p spectra of both catalysts after extreme reoxidation are typical of Ni^2+^ oxidation state (Figure 4). The binding energy (BE) values of the main Ni2p_3/2_ binding energy at 854.0 eV for Ni/BCY15-W-1100 and 853.8 eV for Ni/BCY15-EG-1100 characterize oxidized nickel 2+ state in NiO [47,48,49]. Analysis of the Ni2p peaks by curve fitting undoubtedly displays presence of residual nano-metallic nickel over Ni/BCY15-EG-1100 surface—a weak low-energy peak at 852.3 eV (Figure 4, Table 4) in agreement with the literature data [39,47,50].

**Figure 5:**
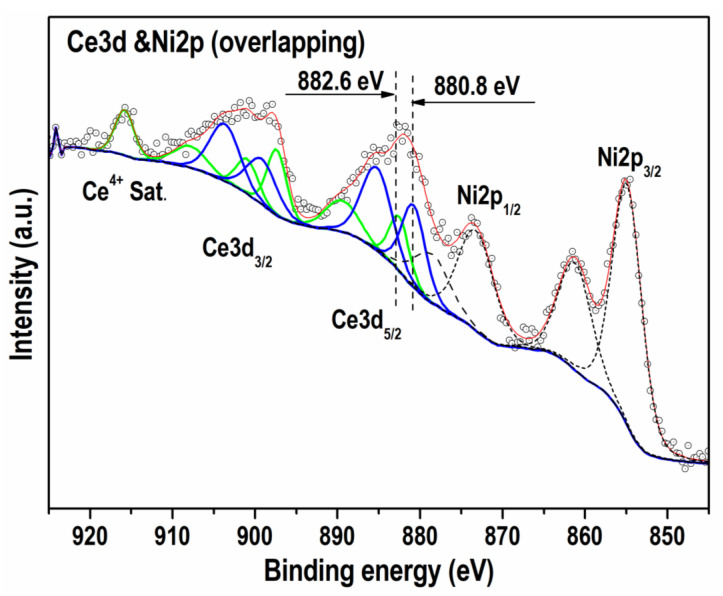
Demonstration of Ce3d and Ni2p lines overlapping.

In contrast to nickel cerium peak is identical with both samples. Moreover, the estimated concentration is the same within the error bars; therefore, we present only one Ce3d spectrum shown in Figure 5. Because of the overlapping peaks, analysis of the Ce3d spectrum is very complex. Ni2p_1/2_ overlaps with Ce3d_5/2_. By using a curve fitting procedure, we were able to distinguish between Ce^3+^ and Ce^4+^ states and estimate their ratio for both investigated samples.

The energy ranges in Figure 5, marked with Ce3d_5/2_, Ce3d_3/2_, and Ce^4+^ Sat., represent the cerium line which is decomposed in two Ce^3+^ (blue line) with typical BE of about 880.8 eV and Ce^4+^ (green line) with BE of about 882.6 eV, respectively [47]. Ce^3+^ and Ce^4+^ ratio was estimated to be 1.5 in favor of Ce^3+^ ions (~60% of total cerium). Dashed line in the curve fitting procedure represents the Ni2p line, which is discussed above.

Overview of the data in Table 4 discloses a tendency of BE values shifting to lower values in the Ni/BCY15-EG-1100 spectra. This observation is valid not only for the main Ni2p_3/2_ peak but also for those of O1s, Ba3d, and Y3d photoelectrons in the spectra of Ni/BCY15-EG-1100 and could be ascribed to different electron densities between atoms compared to Ni/BCY15-W-1100. Based on the equation E_kinetic_ = hν − E_binding_ the interpretation is that the Ni/BCY15-EG-1100 components are characterized by higher kinetic energy. A lower BE value of the Ni^2+^ oxidation state is the indication for a Ni^2+^–O^2−^ bond of moderate strength in the NiO structure which is consistent with the presence of residual nano-metallic Ni particles in this catalyst after reoxidation treatment. Weakening of the bonds of the Ba^2+^ and Y^3+^ ions with oxygen is also an indication.

It should be stated that contrary to other Ni/BCY15-EG-1100 components, Ce3d BEs were registered at higher values (880.9 and 883.5 eV) pointing to a higher degree of interaction between nickel and cerium on the surface relative to Ni/BCY15-W-1100 (880.8 and 882.6 eV). During hydrazine reduction the performance of two simultaneous processes, namely surface reduction of Ce^4+^ to Ce^3+^ by loss of lattice oxygen and Ni interaction with cerium, was confirmed upon analysis of the surface electronic properties of the as-synthesized reduced catalysts noted as Ni/BCY15-W and Ni/BCY15-EG [31]. Using ethylene glycol environment on wet-reduction synthesis it was established that metallic Ni was better stabilized over the partially reduced cerium surface due to a stronger Ni^0^–Ce^3+^ interaction. Higher interaction strength between metallic Ni and Ce^3+^ species was clearly indicated by the position of the Ce^4+^ satellite registered at a higher BE value (916.7 eV) relative to bare BCY15 (916.1 eV) and Ni/BCY15-W (916.3 eV). Obviously, observations for Ce3d region after the reoxidation treatment can also be associated with the existence of Ni^0^ in the Ni/BCY15-EG-1100 structure.

After curve fitting, analysis of O1s, Ba3d, and Y3d regions was also done (Table 4). It can be seen that O anions occur to a less extent on the surface of Ni/BCY15-EG-1100. The O1s level contains two peaks: one at a lower BE due to MeOx structures and owing to oxygen atoms in the lattice of BCY15 whit formula BaCe_0_._85_Y_0_._15_O_2_._925_ [31]. Higher BE values characterize oxygen vacancies (Ox-) registered at 529.9–531.1 eV [51,52,53,54]. Oxygen atomic concentration due to Me-Ox bonds was lower by 10% with Ni/BCY15-EG-1100, which indicates a lower degree of reoxidation. In addition, oxygen vacancy amount was higher by 5%, thus suggesting more surface defects being nucleation sites for higher reactivity of the Ni/BCY15-EG-1100 surface. These findings are further indirect evidence for Ni^0^ presence.

Ba3d photoelectrons can be attributed to Ba–O in the BCY15 lattice (BaCe_0_._85_Y_0_._15_O_2_._925_) and formation of BaCO_3_ during the hydrazine reduction synthesis mainly on using aqueous medium [31]. Appearance of Y_0_._1_Ce_0_._9_O_1_._95_ parasitic phase on extreme thermal reoxidation is shown by aforementioned PXRD results. Y3d curve fitting discloses existence of two anion neighbors of yttrium, Y-O at a lower BE and Y-CO_3_ at a higher BE (Table 4). Reference data about Ba3d and Y3d levels of BCY15 are missing in the current literature. The analysis was based on Ba^2+^ binding energy and Y^3+^ oxidation state [55].

The differences between Ba and Y surface concentrations with both Ni/BCY15-W-1100 and Ni/BCY15-EG-1100 samples (Table 4) are directed to redistribution on the BCY15 surface. A higher nickel dispersion after Ni surface oxidation to NiO was found after evaluation of the Ni/(Ba+Ce+Y) ratio: 1.53 vs. 1.58 for Ni/BCY15-W-1100 and Ni/BCY15-EG-1100, respectively. This indicates a lower nickel surface area for Ni/BCY15-W-1100. This finding combined with evidence for residual nanometallic nickel and better nickel-cerium interface interaction on the Ni/BCY15-EG-1100 surface can potentially affect Ni/BCY15-EG electrode performance.

### 3.5. Transmission Electron Microscopy (TEM)

BCY15 microstructures, as-synthesized reduced Ni/BCY15-W and Ni/BCY15-EG samples, and reoxidized Ni/BCY15-W-1100 and Ni/BCY15-EG-1100 samples were examined by transmission electron microscopy (TEM). Phase composition was registered by selected area electron diffraction (SAED). A careful analysis of selected HRTEM images was carried out to gain more insight into existing phases at the sample surface.

First, TEM measurements of bare BCY15 were performed as a reference. Figure S1 shows a TEM image at magnification of 40,000× (a) and SAED (b). SAED pattern indicates the presence of orthorhombic BaCe_0_._9_Y_0_._1_O_2_._95_ (a = 8.77400 Å, b = 6.23900 Å, c = 6.20400 Å, COD # 96-152-6754). A measured d spacing of 2.66 Å in the HRTEM images presented in sections (c) and (d) corresponds to the (121) plane of BCY in agreement with PXRD data.

TEM (Figure S2a), SAED (Figure S2b), and HRTEM (Figure S2c,d) images of Ni/BCY15-W cermet obtained in aqueous medium provided information for availability of cubic Ni^0^ (a = 3.52414 Å, COD # 96-151-2527) and hexagonal Ni(OH)_2_ (a = 3.11400 Å c = 4.61700 Å, COD # 96-101-1135) phases. The presence of BCY and metallic Ni particles was revealed by measuring interplanar distances of 1.63 Å of the exposed crystal plane (313) and 2.03 Å for (111). These findings are in accordance with PXRD data. However, no crystal phase of Ni(OH)_2_ was detected by PXRD. Its presence on the surface was confirmed by XPS analysis [31]. The phase composition of Ni/BCY15-EG cermet prepared in ethylene glycol was identical (Figure S3). SAED pattern (Figure S3b) and HRTEM (Figure S3c,d) images illustrate the coexistence of BCY, metallic Ni, and Ni(OH)_2_ phases.

A comparative analysis of both Ni/BCY-W-1100 and Ni/BCY-EG-1100 cermets demonstrated a significant effect of forced reoxidation on sample morphology.

**Figure 6:**
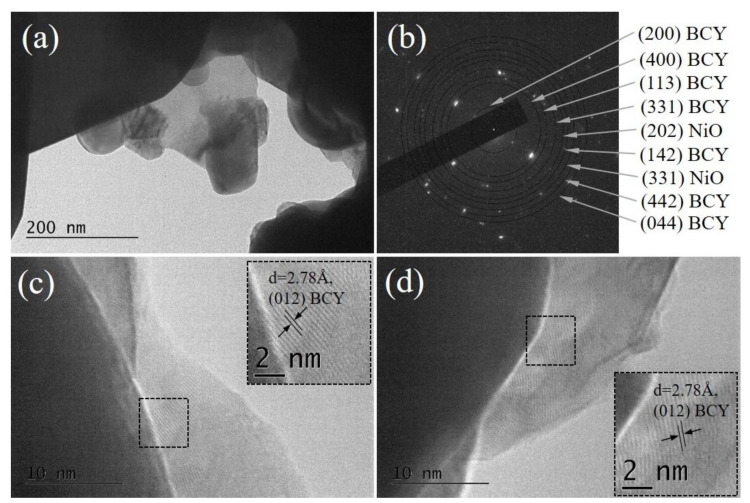
Microstructure of Ni/BCY-W-1100 sample: (**a**) TEM image at magnification 40,000×; (**b**) SAED pattern; (**c**,**d**) HRTEM images at magnification 600,000× with zoomed square areas as insets for better illustration of the lattice fringes.

**Figure 7:**
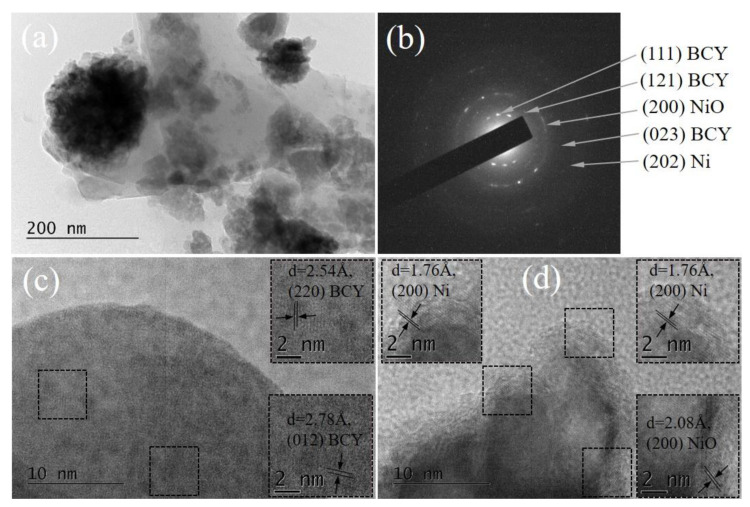
Microstructure of Ni/BCY-EG-1100 sample: (**a**) TEM image at magnification 40,000×; (**b**) SAED pattern; (**c**,**d**) and HRTEM images at magnification 600,000× with zoomed square areas as insets for better illustration of the lattice fringes.

As shown in Figure 6, the Ni/BCY-W-1100 sample exhibits very dense and well-crystallized particles in the shape of a parallelepiped. In contrast, small spherical shaggy particles are observed in Figure 7 where a TEM image of Ni/BCY-EG-1100 is presented. Apparently, sample morphology is closely related with the type of working media. SAED patterns (Figure 6b and Figure 7b) provide further information to support different phase composition, which resulted from the use of deionized water or ethylene glycol. Only cubic NiO (a = 4.16840, COD # 96-101-0094) was registered in the SAED pattern of Ni/BCY-W-1100 sample.

In the case of Ni/BCY-EG-1100 NiO was detected according to the crystal lattice fringes with interplanar distance of 2.08 Å (Figure 7b,d). Additionally, based on performed measurements and HRTEM images, formation of metallic Ni particles was confirmed (Figure 7d). This finding ensures experimental evidence for stabilization of residual metallic Ni particles.

### 3.6. Temperature-Programmed Reduction (TPR)

Hydrogen temperature-programmed reduction was used as a tool for evaluation of the reduction properties of Ni/BCY15 anode catalysts after extreme reoxidation at 1100 °C in air atmosphere. The strength of the chemical interaction between BCY15 and NiO can be examined by TPR. The higher the interaction is, the more difficult NiO to be reduced is, and consequently, a higher hydrogen reduction temperature peak should be observed [56].

**Figure 8:**
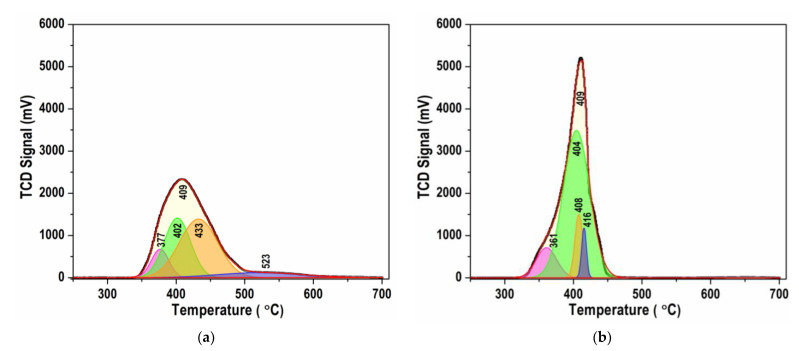
Experimental and simulated differential curves of TPR profiles of reoxidized catalysts: (**a**) Ni/BCY15-W-1100 and (**b**) Ni/BCY15-EG-1100.

Comparison between the TPR profiles shows that both are asymmetric, but some other differences in the reduction temperature range, shapes, and hydrogen consumption values are observed (Figure 8a,b). Reduction of the Ni/BCY-W-1100 sample proceeds within a wide temperature range from 340 to 650 °C (Figure 8a), while the reduction of Ni/BCY-EG-1100 composite occurs in a relatively narrow temperature range of 320–460 °C (Figure 8b). In addition to the main temperature maximum at 409 °C, a shoulder at 450 °C and a broad reduction peak centered at 523 °C characterize the profile of Ni/BCY-W-1100 (Figure 8a).

As is shown in Figure 8b, the TPR profile of Ni/BCY-EG-1100 comprises a single reduction peak at 409 °C, a lower temperature shoulder (360 °C), and a weakly expressed shoulder at 430 °C. The obtained results show a different strength of interaction between BCY15 and NiO: stronger with Ni/BCY15-W-1100 and weaker with Ni/BCY15-EG-1100. Stronger bonds in Ni/BCY15-W-1100 require a higher temperature for breaking resulting in less hydrogen consumption, and indicating fewer numbers of reduced Ni species.

Deconvolution of the TPR profiles into several peaks was performed for accurate interpretation. The obtained data are collected in Table 5. It is seen that in the temperature interval 360–380 °C the reduced part of both samples is similar of about 11%. However, as the temperature increases, the differences in the reducibility of the two samples become significant. While at 402 °C only 33.1% are reduced with Ni/BCY-W-1100, the reduction of Ni/BCY-EG-1100 at the same temperature of 404 °C was dominant to attain 74%. A reduction profile of Ni/BCY-EG-1100 includes two small components at 408 and 416 °C by 9.6 and 4.5%, respectively. In contrast, the reduction of Ni/BCY-W-1100 proceeds at a higher degree of 47.2% at 433 °C followed by high-temperature reduction at 532 °C within 9.2%. This finding is ascribed to reduction of the parasitic BaNiO_2_ phase, which was detected by PXRD study. The shape of the TPR profiles, narrow and stronger with Ni/BCY-EG-1100, and wider and weaker with Ni/BCY-W-1100, testifies the occurrence of different Ni-O bonds by strength in Ni/BCY-W-1100 and almost identical in Ni/BCY-EG-1100.

The existence of strong interaction between hydrazine-originating metallic Ni and Ce^3+^ sites from the BCY15 matrix, by deeper reduction in ethylene glycol-assisted synthesis, was pointed out in our recent paper [31]. A strong Ni^0^–Ce^3+^ bond can be considered a reason for stabilization of Ni^0^ phase during the subsequent high-temperature treatment at 1100 °C.

On the other hand, residual Ni^0^ in Ni/BCY-EG-1100 was established by XPS and HRTEM analysis and very well supported by TPR study. Residual Ni^0^ contributes to creation of sites for dissociative adsorption of hydrogen. These active hydrogen atoms migrate on the surface and reduce Ni^2+^ ions, thus assisting the autocatalytic reduction of NiO and favoring its rapid reduction. This is the reason for performing the reduction of NiO in Ni/BCY-EG-1100 in a narrower temperature range.

### 3.7. Electrochemical Performance

In addition to the structural characterization, the presence of small quantity of Ni phase was observed also electrochemically by electrochemical impedance spectroscopy which is a very sensitive method [57]. The impedances studies were motivated based on previous durability tests of Ni/BCY-EG, applying accelerated stress tests [31]. They were performed by redox cycling of the anode with selected accelerating conditions corresponding to about 2000 h of standard testing [58]. The results obtained in [31] confirmed electrochemically the better long-term performance of the cermet anode obtained in ethylene glycol medium.

**Figure 9:**
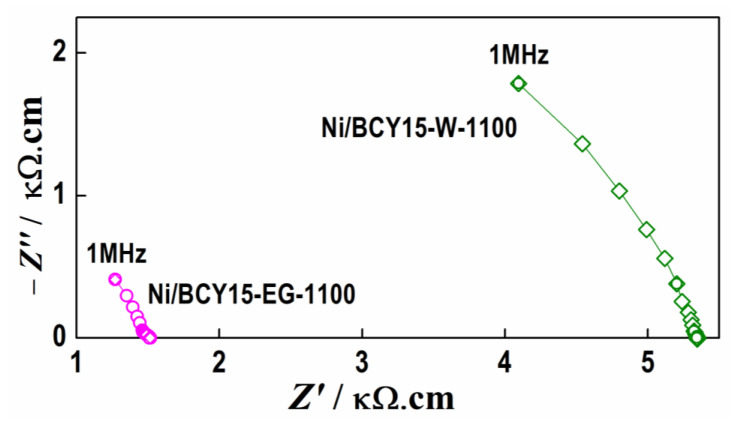
Impedance diagrams of samples Ni/BCY15-W-1100 and Ni/BCY15-EG-1100.

The specially designed impedance experiment in this study aimed to find also electrochemical evidence for the presence of small amounts of metallic Ni in the better performing Ni/BCY-EG-1100 and thus to confirm the results obtained by the characterization tools described in Section 3.1 and Section 3.4, Section 3.5, Section 3.6. Although in small quantities, the presence of pure Ni should decrease the resistance of the cermet. For this purpose, cold pressed samples of the Ni/BCY15-W and Ni/BCY15-EG materials were prepared and heated up to 1100 °C. For improving the contacts with the test rig Pt meshes, Ag current collectors were deposited following a standard procedure, which does not need additional heating. The results are shown in Figure 9. Although having high resistance at room temperature due to the isolating BCY matrix, the sample prepared in ethylene glycol medium has about four times lower resistance. The measurements of the capacitance confirmed similar tendency—lower capacity (20pF) for Ni/BCY15-EG-1100 with respect to Ni/BCY15-W-1100 (38pF) which is also an evidence for the increased conductivity due to the presence of pure Ni in the Ni/BCY15-EG-1100 sample.

## 4. Discussion Summary

Examination of the black color keeping after forced reoxidation upon high-temperature treatment (1100 °C/1 h/air) of as-synthesized reduced Ni/BCY15 catalyst in anhydrous ethylene glycol environment indicates that residual metallic Ni particles undeniably exist in the structure of Ni/BCY15-EG-1100.

Indeed, PXRD technique did not register metallic Ni particles simultaneously with NiO phase. This could be explained by a very low amount of metallic Ni and quite large NiO crystallites of about 350 nm. However, all other applied characterization methods confirmed Ni^0^ presence in the catalyst bulk (HRTEM and TPR) as well as on the catalyst surface (DRS and XPS). TPR profile of Ni/BCY15-EG-1100 supported the existence of metallic Ni sites playing a role in rapid autocatalytic reduction of formed NiO during high-temperature treatment. Surface electronic state by XPS gave evidence for preservation not only of some metallic Ni but also explanation that a stronger Ni^0^–Ce^3+^ bond formed during reduction-synthesis can be considered a reason for some structural stabilization of Ni^0^ phase during the subsequent high-temperature treatment at 1100 °C. It is assumed that Ni^0^ stabilization occurs through bonding to Ce^3+^ sites formed by lattice oxygen transfer founded in a previous investigation of the as-synthesized reduced Ni/BCY15 catalysts [31]. Electrochemical impedance spectroscopy additionally confirmed a lower resistance of Ni/BCY15-EG-1100 due to better conductivity of the cermet by small quantity of residual metallic Ni particles.

It is well-known that metallic nickel is the key factor to adsorption, dissociation, and oxidation of the hydrogen and an electronic conductor that provides electronic conductivity of the anode. Obviously, current evaluation of the stability by applying the hydrazine wet-reduction approach clarifies that established stable bonds between Ni and BCY15 in as-synthesized reduced Ni/BCY15-EG are the reason for the formation of a stable nickel network during the oxidation–reduction cycles as already reported [31] (Figure 7). The new finding for metallic nickel stability is upgraded data regarding degradation of the Ni cermet anode obtained in ethylene glycol medium upon accelerated stress testing. In addition, electrochemical indicators of the cermet obtained in aqueous medium were significantly worse in relation to the cermet synthesized in ethylene glycol environment.

In summary, designing of electrochemical activity of Ni-based anodes by hydrazine wet-reduction methodology provides metallic nickel particles with hindered migration and lower agglomeration degree resulting in high chemical and electrochemical stability of the anode material during operation. Thus, hydrazine wet chemical reduction using ethylene glycol was recognized as a proper approach compared to a commercial mechanical mixing procedure.

Our future research is focused on the improvement of Ni anode stability and long operational lifetime as well as on decreasing the working temperature, which are very important aspects of pSOFC application. Introduction of second component in the Ni/BCY15 system was found to promote higher electronic conductivity, thereby increasing the efficiency of the Ni-based anodic cermet.

## Figures and Tables

**Table 1:** PXRD data of BCY15 and reoxidized catalysts.

Sample	Unit Cell Parameter				Crystal Size L (nm)	ICDD PDF File
	a (Å)	b (Å)	c (Å)	V_cell_ (Å^3^)		
Anode matrix						
BCY15	8.7735 (6)	6.2164 (7)	6.2152 (4)	338.2 (3)	247.3	
Ni/BCY15-W-1100	8.7705 (7)	6.2351 (6)	6.2187 (6)	340.2 (4)	389.1	
Ni/BCY15-EG-1100	8.7728 (4)	6.2343 (3)	6.2183 (3)	340.1 (7)	507.0	
BaCeO_3_	8.7790	6.2360	6.2140	340.19	-	00-022-0074
BaCe_0_._9_Y_0_._1_O_2_._95_	8.7705	6.2393	6.2233	340.55	-	01-081-1386
NiO						
Ni/BCY15-W-1100	4.1792(20)	-	-	72.99(11)	300.0	
Ni/BCY15-EG-1100	4.1791(41)	-	-	72.99(21)	350.0	
NiO	4.1771	-	-	72.88	-	01-070-1429

**Table 2:** Semi-quantitative analysis data of the reoxidized catalysts.

Phase	Sample	
	Ni/BCY15-W-1100	Ni/BCY15-EG-1100
NiO (wt. %)	70.49	70.79
BaCeO_3_ (wt. %)	27.72	29.21
Y_0_._1_Ce_0_._9_O_1_._95_ (wt. %)	1.79	-

**Table 3:** Elemental composition of the reoxidized catalysts.

Sample/Spot	Element Concentration (wt %)				
	Ni	Ba	Y	Ce	NiO
Ni/BCY15-W-1100					
spot 1	60.16	17.88	4.13	17.83	76.56
spot 2	54.29	20.48	4.06	21.17	69.09
spot 3	54.55	20.70	4.09	20.66	69.42
average	56.33	19.69	4.09	19.88	71.68
Ni/BCY15-EG-1100					
spot 1	50.79	22.04	4.42	22.75	64.64
spot 2	48.28	23.07	4.73	23.92	61.44
spot 3	51.78	22.0	4.34	21.88	65.89
average	50.28	22.37	4.49	22.85	63.99

**Table 4:** Surface atomic concentrations and binding energies.

Sample\Element	Ni2p_3/2_		Ce3d		O1s		Ba3d	Y3d	
	Ni^0^	Ni^2+^, NiO	Ce^3+^	Ce^4+^	MeO_x_	Oxygen Vacancies	Ba-O/Ba-CO_3_	Y-O	Y-CO_3_
Ni/BCY15-W-1100									
Total concentration (at. %) (MgKα_Ce3d)	19.60		4.61		64.79		6.38	1.80	
Detailed concentration (at. %)	−	19.60	2.81	1.9	33.04	31.75	6.38	0.85	0.95
BE, eV	−	854.0	880.8	882.6	529.3	531.6	780.0	157.0	158.2
Ni/BCY15-EG-1100									
Total concentration (at. %) (MgKα_Ce3d)	20.24		4.66		63.20		5.28	2.38	
Detailed concentration (at. %)	0.41	19.83	2.8	1.86	29.70	33.5	5.28	0.31	2.37
BE (eV)	852.3	853.8	880.9	883.5	529.2	531.5	779.8	156.5	158.0

**Table 5:** TPR data after profile deconvolution.

Ni/BCY15-W-1100		Ni/BCY15-EG-1100	
T_max_ (°C)	Part (%)	T_max_ (°C)	Part (%)
377	10.5	361	11.7
402	33.1	404	74.2
433	47.2	408	9.6
523	9.2	416	4.5

## Data Availability

The data presented in this study are available in the article.

## Supplementary Materials

The following supporting information can be downloaded at: https://www.mdpi.com/article/10.3390/nano13111781/s1, Figure S1. TEM image at magnification 40,000× (a), SAED (b) and HRTEM image at magnification 400,000× with zoomed square area as inset (c) and 600,000× (d) for BCY sample. Figure S2. TEM image at magnification 40,000× (a), SAED (b) and HRTEM images at magnification 600,000× with zoomed square area as insets (c,d) for Ni/BCY-W sample. Figure S3. TEM image at magnification 40,000× (a), SAED pattern (b) and HRTEM images at magnification 600,000× with zoomed square area as insets (c,d) for Ni/BCY-EG sample.
