# Supplementary material for: Environmentally Benign pSOFC for Emissions-Free Energy: Assessment of Nickel Network Resistance in Anodic Ni/BCY15 Nanocatalyst
Source: Nanomaterials (Basel). 2023 May 31;13(11):1781. doi: 10.3390/nano13111781 (PMC10254282; doi:10.3390/nano13111781)
Supplement: Supplementary file 1 [file nanomaterials-13-01781-s001.zip › nanomaterials-2357610-supplementary.pdf]

Supplementary Materials

Environmentally Benign pSOFC for Emissions-Free Energy: Assessment of Nickel Network Resistance in Anodic Ni/BCY15 Nanocatalyst

Margarita Gabrovska [1], Dimitrinka Nikolova [1,*], Hristo Kolev [1], Daniela Karashanova [2], Peter Tzvetkov [3], Blagoy Burdin [4], Emiliya Mladenova [4], Daria Vladikova [4] and Tatyana Tabakova [1,*]

[1] Institute of Catalysis, Bulgarian Academy of Sciences, 1113 Sofia, Bulgaria; margo@ic.bas.bg (M.G.); hgkolev@ic.bas.bg (H.K.)
[2] Institute of Optical Materials and Technologies, Bulgarian Academy of Sciences, 1113 Sofia, Bulgaria; dkarashanova@yahoo.com
[3] Institute of General and Inorganic Chemistry, Bulgarian Academy of Sciences, 1113 Sofia, Bulgaria; tzvetkov@svr.igic.bas.bg
[4] Academician Evgeni Budevski Institute of Electrochemistry and Energy Systems, Bulgarian Academy of Sciences, 1113 Sofia, Bulgaria; b.burdin@iees.bas.bg (B.B.); e_mladenova@iees.bas.bg (E.M.); d.vladikova@iees.bas.bg (D.V.)
* Correspondence: dimi@ic.bas.bg (D.N.); tabakova@ic.bas.bg (T.T.); Tel.: +359-2-979-3578 (D.N.); +359-2-979-2528 (T.T.)

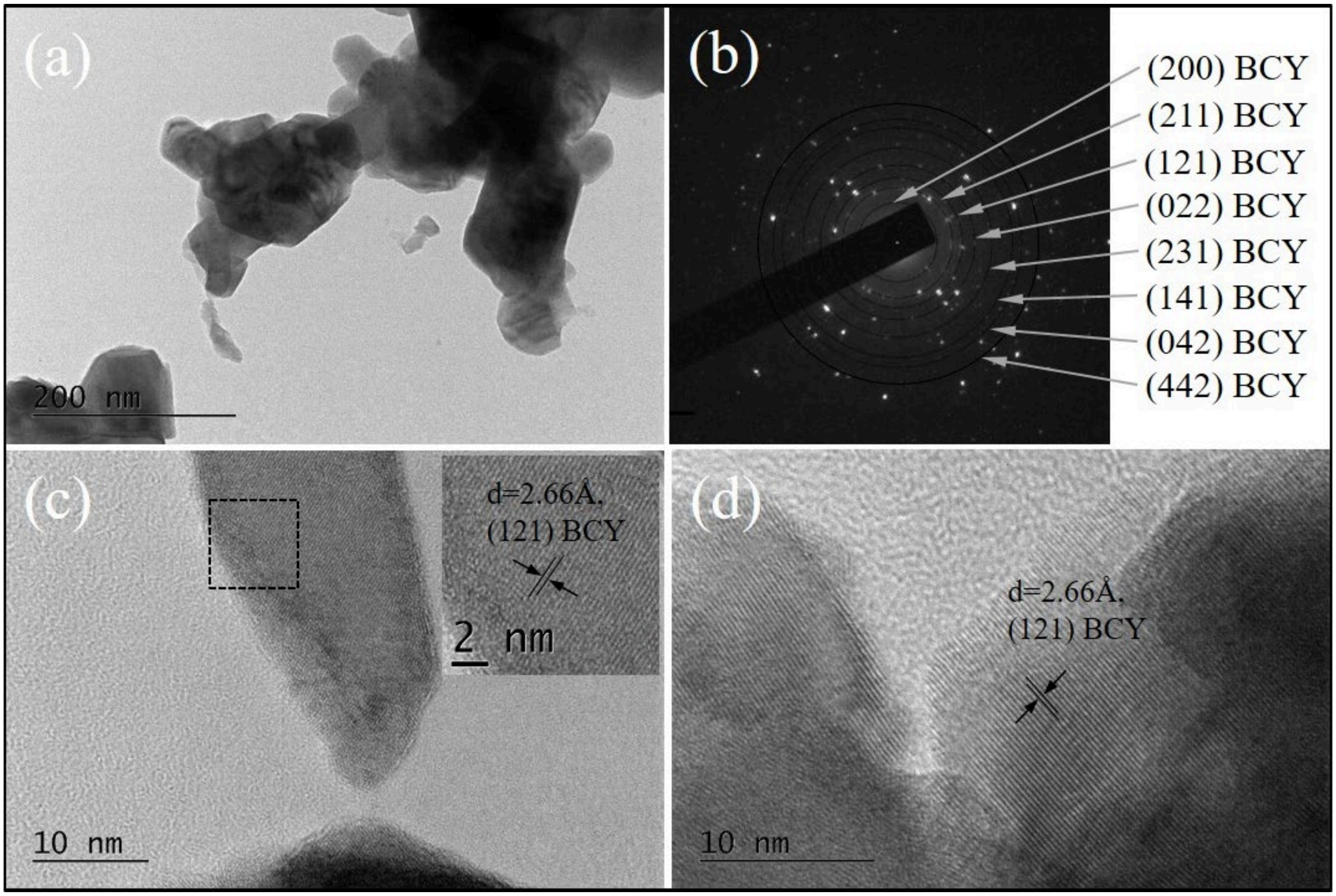

Figure S1. TEM image at magnification 40,000× (**a**), SAED (**b**) and HRTEM image at magnification 400,000× with zoomed square area as inset (**c**) and 600,000× (**d**) for BCY sample.

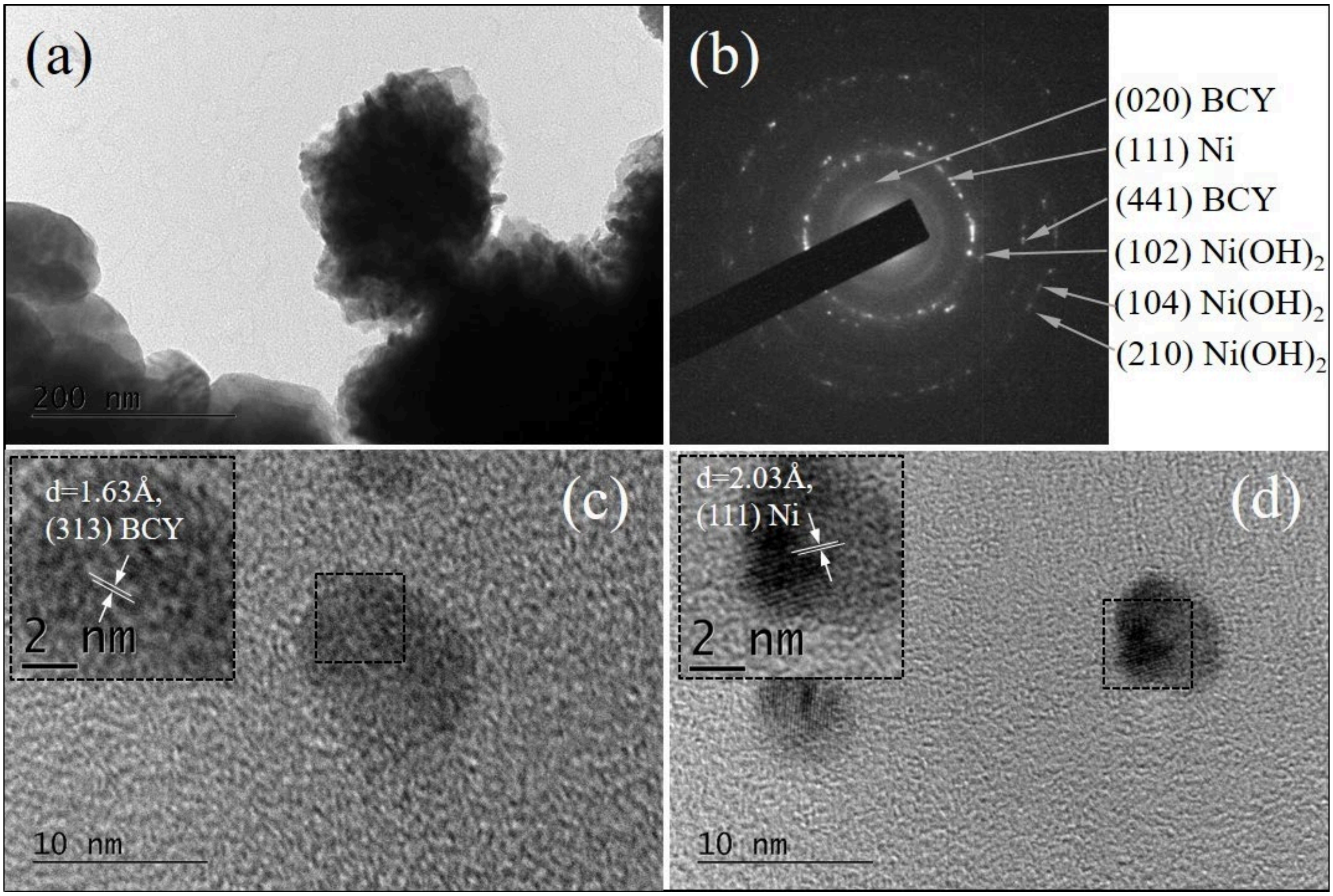

Figure S2. TEM image at magnification 40,000× (**a**), SAED (**b**) and HRTEM images at magnification 600,000× with zoomed square area as insets (**c, d**) for Ni/BCY-W sample.

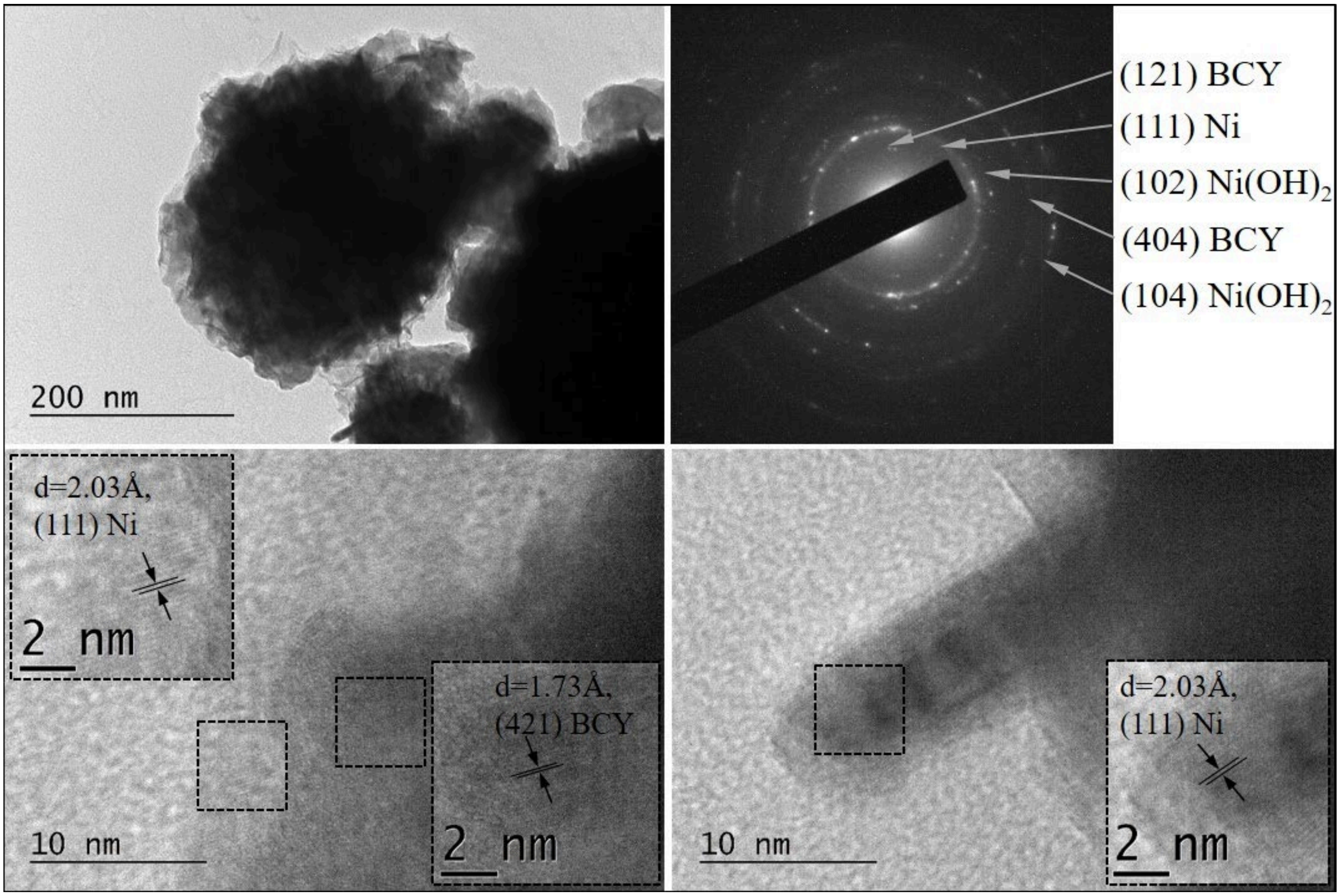

Figure S3. TEM image at magnification 40,000× (**a**), SAED pattern (**b**) and HRTEM images at magnification 600,000× with zoomed square area as insets (**c, d**) for Ni/BCY-EG sample.
